# An Integrated Efficacy and Safety Analysis of Single-Dose Secnidazole 2 g in the Treatment of Bacterial Vaginosis

**DOI:** 10.1007/s43032-019-00048-x

**Published:** 2020-01-01

**Authors:** Helen Pentikis, Nikki Adetoro, Diane Tipping, Sharon Levy

**Affiliations:** 1 Symbiomix Therapeutics, LLC, 1101 E 33rd St, Suite E306, Baltimore, MD 21218 USA; 2 Prosoft Clinical, Wayne, PA USA

**Keywords:** Bacterial vaginosis, Secnidazole, Single-dose treatment

## Abstract

Bacterial vaginosis (BV) is the most common gynecologic infection in women aged 14 to 49 years. Currently recommended treatments require extended dosing and are thus associated with poor adherence. A single-dose oral granule formulation of secnidazole 2 g (SOLOSEC™ [secnidazole], Symbiomix Therapeutics, a Lupin company, Baltimore, MD), a 5-nitroimidazole antibiotic with antimicrobial activity, has been approved by the US Food and Drug Administration for the treatment of BV in adult women. As part of the US registration package, two randomized, double-blind, placebo-controlled clinical studies were conducted to confirm the efficacy and safety of a novel single-dose oral formulation of secnidazole 2 g. This is an integrated analysis of efficacy and safety results from these studies, pivotal study 1 and pivotal study 2. By combining the results of the two studies, relevant information is presented especially when considering the effect of secnidazole on patients with recurrent episodes of BV and the difference in effect on patients of black race. Single-dose secnidazole 2 g was statistically superior to placebo on all primary and secondary efficacy outcomes in both trials, including clinical outcome responder rate (*P* < 0.001), achievement of Nugent scores in the normal range of 0 to 3 (*P* < 0.001), greater numbers of patients as therapeutic outcome responders at the test of cure/end of study visit on days 21–30 (*P* < 0.001), and fewer patients requiring additional treatment at the test of cure/end of study visit (*P* < 0.001), supporting the role for single oral dose secnidazole 2 g granules as treatment for women with BV.

## Introduction

Every year, upwards of 21 million females between the ages of 14 and 19 years are affected by bacterial vaginosis (BV), making it the most common gynecologic infection in women in this age group [1, 2]. Symptoms are associated with a two-fold increase in risk of complications such as acquisition of the sexually transmitted infections (STIs), including human immunodeficiency virus (HIV), herpes simplex type 2 virus, *Neisseria gonorrhoeae*, and *Chlamydia trachomatis.* Furthermore, preterm delivery and premature membrane rupture are more likely to affect pregnant women who have BV [2, 3].

The current standard of care (SOC) treatments require extended dosing, such as 7 days of twice-daily oral metronidazole (500 mg), 5 days of intravaginal metronidazole (0.75% gel), or 7 days of intravaginal clindamycin (2.0% cream) (Table 1) [4, 5]. Poor adherence, which increases with longer durations of treatment and more complex care paradigms, is therefore common. Studies of compliance rates have found that nearly 50% of patients do not adhere to a 5- to 7-day treatment regimen [6, 7]. Low patient compliance leads to failure of treatment, recurring infections, and, possibly, accelerated growth of drug-resistant microorganisms [8]. Poor adherence to the current BV SOC drug regimens may therefore be a contributing factor to the increasing incidence of chronic BV and the need for multiple antibiotic treatments in an individual patient [4, 9].

**Table 1:** Current recommended BV treatment regimens

Therapy	Formulation	Administration		Duration
First-line therapy				
Metronidazole	500 mg	2× daily	Orally	7 days
Metronidazole	0.75% gel	1× daily	Intravaginally	5 days
Clindamycin	2.0% cream	1× daily	Intravaginally	7 days
Alternative therapies				
Tinidazole	2 g	1× daily	Orally	2 days
Tinidazole	1 g	1× daily	Orally	5 days
Clindamycin	300 mg	2× daily	Orally	7 days
Clindamycin	100 mg	1× daily	Intravaginally	3 days

Adapted from Workowski KA, et al., 2015^4^

SOLOSEC™ (secnidazole) oral granules, a novel 2-g granule formulation of secnidazole that requires a treatment regimen of only one oral dose, has recently been approved by the US Food and Drug Administration (FDA) for the treatment of BV in adult women. A 5-nitroimidazole, secnidazole, has been shown in vitro to have antimicrobial properties against a number of anaerobic gram-positive and gram-negative bacteria, though not against *Lactobacillus* [10, 11]. Evidence of the clinical and microbiologic activity of secnidazole has been examined in controlled, multicenter studies in patients with BV [10]. Furthermore, single-dose secnidazole 2 g has a favorable safety profile, is free of drug interactions, and does not have an alcohol restriction like other nitroimidazoles. The pharmacokinetics of secnidazole is characterized by a prolonged half-life (17 h compared with 8 h for metronidazole) and sustained drug exposure. The single oral dose regimen is simple, and it is administered by mixing it with soft foods such as pudding, applesauce, or yogurt. Together, these features are likely to increase patient adherence and show improved clinical outcomes in the treatment of BV.

A number of clinical trials explored the safety and efficacy of secnidazole prior to its approval by the FDA. Five phase 1 studies in healthy volunteers evaluated its clinical pharmacology and informed the labeling of SOLOSEC™, including dose administration, the absence of a food effect on administration, and the absence of an interaction with alcohol [12–17]. To explore clinical outcomes, two randomized, double-blind, placebo-controlled clinical studies, one dose-ranging phase 2 study (pivotal study 1) [18] and one phase 3 study (pivotal study 2) [19], were conducted to confirm its efficacy and safety. Here, an integrated analysis of efficacy and safety results from the pivotal studies 1 and 2 combined is presented.

## Materials and Methods

Pivotal studies 1 and 2 were similar in design and scope. Both studies were multicenter, prospective, randomized, double-blind, and placebo-controlled. Pivotal study 1 was a dose-ranging study that included 215 nonpregnant women 18 years or older across 24 centers in the USA. A single oral dose of 1 g or 2 g, randomized 1:1:1 with placebo, was administered. Pivotal study 2 included 189 women/postmenarchal girls across 21 centers in the USA. A single oral dose of secnidazole 2 g, randomized 2:1 with matched placebo (*n* = 125:64), was administered. The selection criteria of both studies required that patients meet all four Amsel criteria for BV, consistent with the FDA guidance for BV: an abnormal discharge, pH ≥ 4.5, ≥ 20% clue cells, and a positive 10% potassium hydroxide (KOH) whiff test [20]. In both studies, patients were stratified by the number of self-reported episodes of BV, including the current episode, in the past 12 months, and pivotal study 2 was also stratified by race (black vs all others) (Table 2). Secnidazole treatment was self-administered on day 1 (without regard to meals) in both studies and patients were to return for a “test of cure” (TOC) visit between days 21 and 30 or an “end of study” (EOS) visit if the final visit occurred before day 21 or after day 30. In pivotal study 1, patients also returned to the site for one interim visit between days 7 and 14.

**Table 2:** Integrated phase 2 and phase 3 study patient demographics in the mITT population

Parameter	Secnidazole 2 g (studies 201/301) (*N* = 169)	Placebo (studies 201/301) (*N* = 119)
Age (years)		
*n*	169	119
Mean (SD)	33 (8.9)	31 (7.8)
Median	31	31
Min, max	18, 54	18, 49
Gender, (*n*** (%))**		
Female	169 (100)	119 (100)
Ethnicity, (*n*** (%))**		
Hispanic or Latino	27 (16)	17 (14.3)
Not Hispanic or Latino	142 (84)	102 (85.7)
Race, (*n*** (%))**		
White	78 (46.2)	50 (42)
Black or African American	85 (50.3)	63 (52.9)
Asian	1 (0.6)	3 (2.5)
American Indian or Alaska Native	1 (0.6)	0
Native Hawaiian or Other Pacific Islander	0	0
Other	4 (2.4)	3 (2.5)
Race Strata, (*n*** (%))**		
Black	85 (50.3)	63 (52.9)
All others	84 (49.7)	56 (47.1)
Number of BV episodes in the past 12 months		
*n*	169	119
Mean (SD)	3 (2.6)	3 (2.3)
Median	2	2
Min, max	1, 12	1, 12

*SD* standard deviation

The study population used for the integrated efficacy analysis was the modified intent-to-treat (mITT) population (*n* = 288: 169 secnidazole, 119 placebo), which included patients who were randomized and met all inclusion/exclusion criteria. Both studies were performed in keeping with local legal requirements. Before the start of the study, the study protocol and other appropriate documents were submitted to the institutional review board (IRB) (Schulman Associates IRB, Cincinnati, OH; Western IRB, Puyallup, WA).

The integrated efficacy analysis combined the efficacy data for 2-g secnidazole vs placebo from both studies. The primary endpoint was the clinical outcome responder (COR) rate (clinical cure) at the TOC or EOS visit. To be considered a clinical responder, the patient must have achieved normalization of discharge amine odor and clue cells, a negative whiff test, and a clue cell population that was < 20% of the total epithelial cells present upon microscope examination. A patient achieving therapeutic outcome was defined as a patient achieving the COR criteria and having a Nugent score of 0 to 3.

The Cochran-Mantel-Haenszel statistical test, stratified by study, BV history (3 or fewer vs 4 or more episodes in the past 12 months), and race (black vs all others), was used to compare the primary endpoint outcomes for secnidazole 2 g vs placebo. Statistical significance was defined a priori at *P* < 0.05 (two-sided). A 95% binomial confidence interval (CI) of the responder rate within treatment was calculated.

Clinical BV symptomatology was assessed in each individual study. Although the integrated analysis did not specifically analyze the impact a single 2-g dose of secnidazole had on symptoms early on, each individual pivotal study did assess BV symptom relief. In pivotal study 1, an exploratory efficacy analysis was performed, in which patients were asked to complete a daily telephone questionnaire on days 2 through 7 postdose to assess BV symptom relief [18, 21]. Similar vaginal assessments were performed in pivotal study 2 as part of the secondary efficacy analysis during the interim visit (days 7 to 14, postdose) [19, 22].

The integrated safety analysis combined the safety results from pivotal studies 1 and 2. The safety analysis included all randomized patients who received the study medication. The safety evaluation was based on the incidence, intensity, and types of adverse events (AEs). Also collected but not presented here were changes to vital signs (systolic and diastolic blood pressure, pulse, and body temperature) and clinical laboratory results.

## Results

### Efficacy

In the integrated analysis (assessed in 169 patients receiving 2-g secnidazole and 119 patients receiving placebo) of pivotal studies 1 and 2, the primary outcome measure of COR for 2-g single-dose secnidazole was superior to that of placebo (58.6% for 2-g secnidazole vs 18.5% for placebo, *P* < 0.001) (Table 3). Of patients treated with 2-g secnidazole who had reported three or fewer episodes of BV in the previous year, 64.5% were found to be CORs, as were 42.2% of those who had reported four or more cases of BV in the previous year. Of those treated with placebo, 20.9% of the three or fewer BV strata and 12.1% of the four or more BV strata were CORs. Likewise, the secondary outcome measures of Nugent score, therapeutic outcome, and investigator’s clinical assessment all supported the efficacy of 2-g secnidazole over placebo. Significantly more patients in the 2-g secnidazole treatment group had Nugent scores of 0 to 3, which are considered a normal range, compared with those of the placebo group (42.6% of secnidazole-treated vs 5.9% of placebo-treated, *P* < 0.001). Of patients in the 2-g secnidazole group, 36.7% were found to be therapeutic outcome responders at the TOC/EOS visit (days 21 and 30), compared with 5.0% of placebo group patients (*P* < 0.001). The investigator’s clinical assessment considered whether the patient needed additional BV treatment. Patients in the 2-g secnidazole group were significantly less likely to need an additional treatment by the TOC/EOS visit than those in the placebo group (29.4% of secnidazole vs 72.3% of placebo group patients, *P* < 0.001). When the integrated results were stratified by race, 49.4% of black patients in the 2-g secnidazole group were found to be CORs compared with 67.9% of patients of all other races combined.

**Table 3:** Summary of COR rates in the mITT population

	Secnidazole 2 g (studies 201/301) (*N* = 169), *n* (%)	Placebo (studies 201/301) (*N* = 119), *n* (%)
COR^a^	99 (58.6)	22 (18.5)
Nonresponder^b^	70 (41.4)	97 (81.5)
*P* value^c^	< 0.001	
95% Exact binomial CI for responder rate	50.8, 66.1	12.0, 26.6

^a^*COR* is defined as a patient who had all three of the following clinical assessments: normal vaginal discharge, negative KOH whiff test, and clue cells < 20%

^b^2, 10, and 7 patients in SYM-1219 1 g, 2 g, and placebo, respectively, were missing one or more of the clinical assessments and were classified as nonresponders

^c^*P* value vs placebo from CMH test adjusted for study (201 or 301), BV strata (≤ 3 or > 3 episodes in the past 12 months), and race (black or all others)

### Safety

The integrated safety analysis was assessed in 404 patients receiving 1 or 2-g secnidazole and 136 patients receiving placebo from pivotal studies 1 and 2. Overall, single-dose secnidazole 2 g was well tolerated, with safety outcomes similar to those of placebo. The majority of patients who received treatment completed the study (87.3% (*n* = 172) of those receiving 2-g secnidazole and 82.4% (*n* = 112) of those receiving placebo). No treatment-related serious AEs were reported. One or more treatment-emergent adverse events (TEAEs) were experienced by 28.9% of patients in the 2-g secnidazole group, compared with 15.4% of those in the placebo group (Table 4). Treatment-related TEAEs were reported for 16.2% of patients in the 2-g secnidazole group and 5.9% of those in the placebo group. No deaths occurred.

**Table 4:** Overall summary of TEAEs in the safety population

	Secnidazole 2 g (studies 201/301) (*N* = 197), *n* (%)	Placebo (studies 201/301) (*N* = 136), *n* (%)
Patients with 1 or more TEAE	57 (28.9)	21 (15.4)
Patients with 1 or more treatment-related^a^ TEAE	32 (16.2)	8 (5.9)
Patients with 1 or more severe TEAE	5 (2.5)	0
Patients with 1 or more serious TEAE	2 (1.0)	0
Patients who discontinued from the study due to a TEAE	0	1 (0.7)
Total number of TEAEs	103	25
Total number of treatment-related^a^ TEAEs	60	9
Total number of severe TEAEs	7	0
Total number of serious TEAEs	2	0

^a^Includes possibly and probably related AEs

Common treatment-related TEAEs, occurring in ≥ 2% of secnidazole-treated patients, were vulvovaginal candidiasis (VVC; 9.6%), headache (3.6%), nausea (3.6%), diarrhea (2.5%), abdominal pain (2.0%), and vulvovaginal pruritus (2.0%) (Table 5).

**Table 5:** Common AEs occurring in ≥ 2% of secnidazole-treated patients

Adverse reaction	Secnidazole (*N* = 197), *n* (%)	Placebo (*N* = 136), *n* (%)
VVC	19 (9.6)	4 (2.9)
Headache	7 (3.6)	2 (1.5)
Nausea	7 (3.6)	1 (0.7)
Diarrhea	5 (2.5)	1 (0.7)
Abdominal pain	4 (2.0)	2 (1.5)
Vulvovaginal pruritus	4 (2.0)	2 (1.5)

## Discussion

Secnidazole 2 g is a single-dose, oral granule formulation and is a member of the 5-nitroimidazole family. Based on the clinical experience reported, with secnidazole as an effective treatment for bacterial and parasitic diseases [10, 23], secnidazole has been developed as a treatment for women with BV. The formulation and pharmacokinetics of secnidazole, characterized by a relatively long half-life (~ 17 h) and resultant higher, more sustained plasma concentrations, allow for a single-dose regimen. As a result, the entire treatment course can be completed with the first and only drug dose. This “one and done” single-dosing strategy offers improved patient adherence and holds the promise of improved patient outcomes with a lower incidence of undertreated BV, given that the entire treatment course is completed with just a single oral dose. This dosing regimen is in distinct contrast to the currently used oral treatment regimens requiring extended dosing that are known to impact patient adherence [6, 7].

Pivotal study 1 [18] and pivotal study 2 [19], discussed herein, are the first placebo-controlled clinical trials of single-dose secnidazole 2 g for the treatment of BV, and they were conducted in support of the registration of the drug in the USA. Pivotal study 2 was conducted to confirm the efficacy and safety that had been demonstrated by pivotal study 1. Despite the availability of effective treatments, BV remains an unmet medical need given the prevalence of this infection combined with the variable response rates to currently available therapies [7, 10, 24–27]. The successful conduct of these studies provided the foundation for drug approval in the USA and provides an alternative to the current treatments in the USA that often involve multiday treatments (i.e., oral tinidazole, metronidazole) or require dosage forms that some patients may not prefer or that may have lower success rates than oral products (i.e., intravaginal clindamycin, intravaginal metronidazole).

The results herein support the use of single-dose secnidazole 2 g for the treatment of patients with BV in the USA. Minimal safety issues were encountered. As such, there is only a very short list of AEs, included in the SOLOSEC™ label, that occurred in > 2% of patients. VVC is a common side effect of drugs in the class [28–30] that occurs in a small percentage of patients. In this integrated analysis, 4.1% of patients taking secnidazole 2 g developed VVC, compared with 0.7% of patients taking placebo. This is similar to the results seen in our other studies [19]. In addition, secnidazole 2 g was found to have a minimal metallic taste (data not shown), a common complaint among patients taking nitroimidazoles [31]. This may contribute to the possible improvement in adherence that may be observed in this single-use treatment.

These studies investigated efficacy and safety in a representative and diverse patient population. Racial diversity (> 50% African American) and other demographics were consistent across study groups. Patients included those who had recurrent BV. Because this was a highly affected population, of which more than 40% had had four or more episodes of BV in the previous year, treatment groups were stratified by previous BV episodes. In 2016, new current standards were described by the FDA. Previous studies comparing the efficacy of secnidazole with that of metronidazole may not have met these new, more rigorous standards for assessment [32]. Pivotal study 2, included as part of these integrated results, was designed with these standards in mind and has demonstrated the efficacy of secnidazole.

Rapid symptom relief is an important consideration with BV treatment; therefore, the assessments of BV symptom relief in each individual study are important to note. In pivotal study 1, more patients who achieved a clinical outcome response on the 2-g dose of secnidazole reported normal/not an issue or mildly abnormal clinical symptoms on day 4 postdose, vaginal discharge (67.5% (25/37 responses)) and/or vaginal odor (78.4% (29/37 responses)) [18, 21]. In pivotal study 2, as part of the secondary efficacy analysis, during the interim visit (days 7 to 14, postdose), 71% (69/107) of patients had normal vaginal discharge and 84.5% (82/107) had a negative KOH whiff test [19, 22]. In general, symptom resolution was seen soon after treatment with secnidazole 2 g and the proportion of patients who reported normal vaginal discharge and vaginal odor increased over both study periods.

In summary, the safety and efficacy data generated by the clinical development program demonstrate the clinical benefit of secnidazole 2 g as treatment for women diagnosed with BV. Secnidazole 2 g offers patients a single oral dose treatment that is safe and effective.

## Conclusions

Single-dose secnidazole 2 g was statistically superior to placebo on all primary and secondary efficacy outcomes, and it was well tolerated. These combined findings support the role of SOLOSEC™ oral granules for the treatment of BV in adult women. This single-dose formulation may increase patient adherence to the treatment regimen and improve overall outcomes.
